# Superstatistical analysis and modelling of heterogeneous random walks

**DOI:** 10.1038/ncomms8516

**Published:** 2015-06-25

**Authors:** Claus Metzner, Christoph Mark, Julian Steinwachs, Lena Lautscham, Franz Stadler, Ben Fabry

**Affiliations:** 1 Department of Physics, Biophysics Group, Friedrich-Alexander-Universität Erlangen-Nürnberg (FAU), Erlangen 91052, Germany

## Abstract

Stochastic time series are ubiquitous in nature. In particular, random walks with time-varying statistical properties are found in many scientific disciplines. Here we present a superstatistical approach to analyse and model such heterogeneous random walks. The time-dependent statistical parameters can be extracted from measured random walk trajectories with a Bayesian method of sequential inference. The distributions and correlations of these parameters reveal subtle features of the random process that are not captured by conventional measures, such as the mean-squared displacement or the step width distribution. We apply our new approach to migration trajectories of tumour cells in two and three dimensions, and demonstrate the superior ability of the superstatistical method to discriminate cell migration strategies in different environments. Finally, we show how the resulting insights can be used to design simple and meaningful models of the underlying random processes.

## Abstract (web summary)

Conventional methods to quantify the migratory behaviour of cells assume that underlying parameters are constant. Mark *et al.* apply a superstatistical approach to extract time-dependent parameters of motile cells, and demonstrate an enhanced ability to distinguish between different migration strategies.

## Graphical abstract

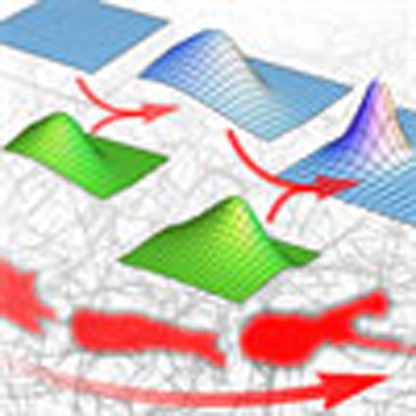

Stochastic time series, here used synonymously with random walks, play an important role in earth- and life sciences, technology, medicine and economics. Most of these disciplines deal with complex systems in which multiple hierarchical processes are interacting at different timescales. Systems with this level of complexity are likely to change their statistical properties as a function of time, resulting in heterogeneous time series. It is therefore surprising that only few tools are available for the analysis and characterization of such time-varying random walks. Some of these tools are used in finance^1^,^2^,^3^, mainly with the goal of forecasting. In science, heterogeneous time series have been successfully described by Hidden Markov models^4^. However, systems with continuously time-varying statistics cannot be adequately modelled by a few discrete hidden states.

Owing to this lack of appropriate tools, many studies are still relying on conventional evaluation methods that were designed for simple physical systems. The most frequently used statistical measures for random walks, in particular the step width distribution (SWD), the mean-squared displacement (MSD) and the velocity autocorrelation function, are implicitly assuming that the stochastic process can be globally described by a few characteristic parameters, such as a constant variance and a constant correlation time.

We demonstrate in this paper that the application of these conventional methods to heterogeneous random walks generates ‘anomalous' results, such as non-Gaussian SWDs or power-law MSDs with fractional exponents^5^,^6^,^7^. These anomalies emerge inevitably from the temporal averaging over changing local statistics during the evaluation period (Supplementary Note 1), and therefore do not provide meaningful insights into the random walk apart from its heterogeneous nature. Moreover, these temporally averaging measures may remain unchanged even if the experimental conditions are significantly altered. This lack of sensitivity points to a fundamental limitation of conventional statistical methods for analysing heterogeneous processes. SWD, MSD and autocorrelation function average over the successive statistical parameters of the heterogeneous random walk, instead of using the parameter dynamics as a rich additional source of information.

In this study, we propose a superstatistical framework for modelling and analysing heterogeneous random walks. The term superstatistics refers to the superposition of several different stochastic processes^8^,^9^,^10^,^11^. Accordingly, we describe the time series locally by a homogeneous random walk model with a minimum number of statistical parameters. In the case of cell migration, we use an autoregressive process of first order (AR-1) with a persistence parameter *q* and an activity parameter *a*. These parameters (*q*_*t*_,*a*_*t*_) are allowed to change with every time step of the random walk. By this way, heterogeneous time series of arbitrary complexity can be described (Supplementary Note 2).

We provide a new sequential Bayesian method to infer the time-dependent parameters from measured random walk trajectories. In contrast to conventional maximum likelihood parameter estimation within a sliding time window, our method can handle both gradual and abrupt changes of the parameters. As a Bayesian method, it provides not only point estimates but also their confidence intervals. After extraction of (*q*_*t*_,*a*_*t*_) from the measurements, the statistical properties of the time-dependent parameters can be subsequently analysed by computing the temporally averaged joint posterior distribution *p*(*q*,*a*), the temporal auto-correlations *C*_*qq*_(Δ*t*) and *C*_*aa*_(Δ*t*), and the cross-correlations *C*_*qa*_(Δ*t*).

In this paper, we use the migration of individual tumour cells as a case study of superstatistical analysis. Cell migration plays an essential role in many fundamental biological processes, such as embryogenesis, tissue repair or cancer development^12^,^13^,^14^. Anomalous features of cellular random walks have been reported by several groups, and a variety of models have been proposed in the literature to account for those anomalies^5^,^7^,^15^,^16^,^17^,^18^.

We demonstrate that anomalies of conventional statistical measures to describe cell migration are attributable to fluctuations of migration persistence *q* and activity *a*. Moreover, the joint distribution of persistence and activity, *p*(*q*,*a*), and the auto- and cross-correlations *C*_*ij*_(Δ*t*) of these two parameters provide characteristic fingerprints of the underlying random walks. Unlike globally averaging statistical measures, a superstatistical analysis can clearly resolve the effects of different environments on cell migration, such as migration in a three-dimensional (3D) collagen network versus migration on a planar 2D culture dish. Furthermore, by observing individual cells in microfabricated 1D channel structures with varying diameter, we demonstrate that the temporal changes of the (*q*_*t*_,*a*_*t*_)-parameters are directly associated with different local microenvironments that the cells experience along their migration path. Finally, we show how the extracted statistical properties of the time-dependent parameters can be used to construct simplified models that reproduce all key features of the data, including the non-Gaussian SWD and power-law MSD. While other types of models have also successfully reproduced these anomalous features, for example, using fractional diffusion equations^7^ or integro-differential equations with complex memory kernels^19^, the superstatistical framework achieves this with the simplest persistent random walk model (the two-parameter AR-1 process), extended by the temporal variations of the two parameters (persistence and activity).

## Results

### Cell migration in 2D and 3D

**Figure 1:**
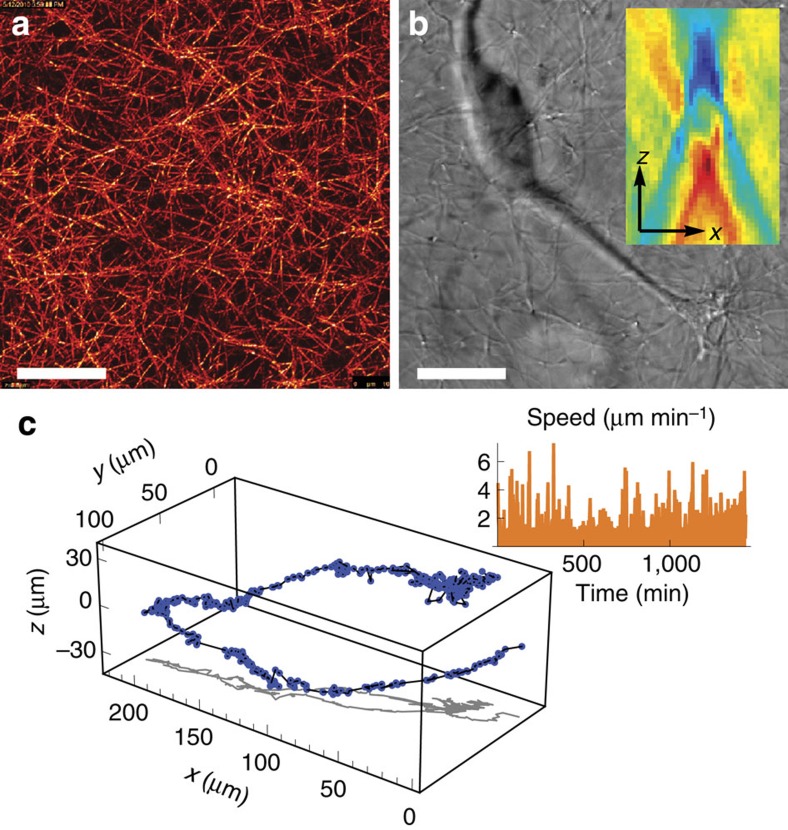
Tracking and analysis of cells migrating in 3D collagen networks. (**a**) Confocal image of a collagen gel. (**b**) Bright-field image of an MDA-MB-231 breast carcinoma cell that has migrated into the bulk of the collagen to a depth of 200 μm. Scale bars, 20 μm. Inset: the characteristic light intensity profile (*z*–*x* plane) around the cell nucleus is used to track the cell position within the gel with an accuracy of 2 μm (r.m.s.). (**c**) Example of a 3D cell trajectory, sampled at 2.5 min time intervals. Inset: momentary speed as a function of time.

We study the migration of the breast carcinoma cell line MDA-MB-231 in a 3D collagen gel and on a tissue culture-treated 2D plastic surface, either uncoated and or coated with the adhesion ligand fibronectin. Three-dimensional cell positions within the random fibre network of a collagen gel (Fig. 1a,b) are detected by analysing the characteristic refraction pattern (Fig. 1b inset) around the cell nucleus. From the individual cell trajectories (Fig. 1c), we compute momentary migration properties, such as cell speed versus time (Fig. 1c inset). Since the gel has a free upper surface and thus a lower effective stiffness in the *z*-direction, cells react with a more pronounced horizontal (*x*–*y* direction) alignment and motion, in agreement with theoretical predications based on active cellular mechanosensing mechanisms^20^. Therefore, only the *x*–*y* coordinates are used for comparing 2D and 3D migration.[Fig f2][Fig f3][Fig f4]

### Globally averaging statistical measures

For each individual cell trajectory, we compute the SWD, defined as the probability *p*(Δ*x*,Δ*t*) that the cell changes its *x*-coordinate by Δ*x* within a lag time interval Δ*t*, as well as the MSD, defined as *r*^2^(Δ*t*)=〈(**r**(*t*+Δ*t*)−**r**(*t*))^2^〉_*t*,*e*_, where 〈〉_*t*,*e*_ indicates temporal and subsequent ensemble averaging over the different individual cells of the same migration environment.

Regardless of environment, the SWD shows a leptocurtic, approximately exponential shape (Fig. 5a inset and Supplementary Note 3). For lag times below 500 min, the MSD can be approximated by power laws (Fig. 5a) with a fractional exponent of 1.3 in the cases of 3D collagen and uncoated 2D plastic, but with a larger exponent of 1.7 in the case of fibronectin-coated 2D plastic. It is remarkable that the SWD and MSD are practically indistinguishable for migration in 3D collagen and on uncoated 2D plastic, even though these environments require different migration strategies.

Within collagen, cells assume a pronounced elongated shape and typically form a path-finding long and thin protrusion that can extend over >100 μm (Supplementary Movies 1 and 2; ref. 21). The directionally persistent trajectory of the cells is mainly defined by the contour of this long protrusion, resembling the movement of a needle in an array of obstacles^22^. However, cells can also pull themselves along bundles of collagen fibres in a process known as contact guidance^23^,^24^. Occasionally, encounters with obstacles or small pores in the disordered collagen network can force the cell to withdraw or change directions (Supplementary Movie 2). On planar surfaces by contrast, the cells spread and assume a flat, irregular shape. They also polarize and move preferentially along their polarization axis (Supplementary Movie 3), but they cannot take advantage of external cues to keep a persistent migration direction.

Despite these diverging migration modes, the net spatial advancement of MDA-MB-231 cells over time is similar in both environments. Therefore, the SWD and MSD for migration in 3D collagen and on uncoated 2D plastic are nearly identical. On fibronectin-coated 2D plastic, the cells migrate more slowly but with a higher directional persistence (Supplementary Movie 4). Over time, this leads to a larger net spatial advancement compared with uncoated plastic. Accordingly, the MSD shows a higher fractional exponent of 1.7, and the SWD broadens (Fig. 5a).

### Bayesian inference of time-dependent parameters

For the superstatistical analysis of the data, we first compute for each cell trajectory {**r**_*t*_=(*x*_*t*_,*y*_*t*_)} the vectorial displacements **u**_*t*_=**r**_*t*_−**r**_*t*−1_ for each measurement time step *δt*=5 min. The statistical relationship between two successive displacements is described by a 2D first-order autoregressive process (AR-1) defined by


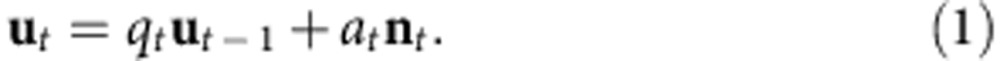


This process is equivalent to a persistent random walk or a time-discrete Ornstein–Uhlenbeck process. The parameter *q*_*t*_∈[−1,+1] describes the local persistence of the random walk, with *q*_*t*_=−1 corresponding to anti-persistent motion, *q*_*t*_=0 to non-persistent diffusive motion and *q*_*t*_=+1 to persistent motion. The parameter *a*_*t*_∈[0,∞] describes the local activity (noise amplitude) and sets the spatial scale of the random walk. Together, the two parameters determine the variance of the displacements according to var(*u*)=*a*^2^/(1−*q*^2^). The vector ***n***_*t*_=(*n*_*xt*_,*n*_*yt*_) is normally distributed, uncorrelated random noise with unit variance.

**Figure 2 f2:**
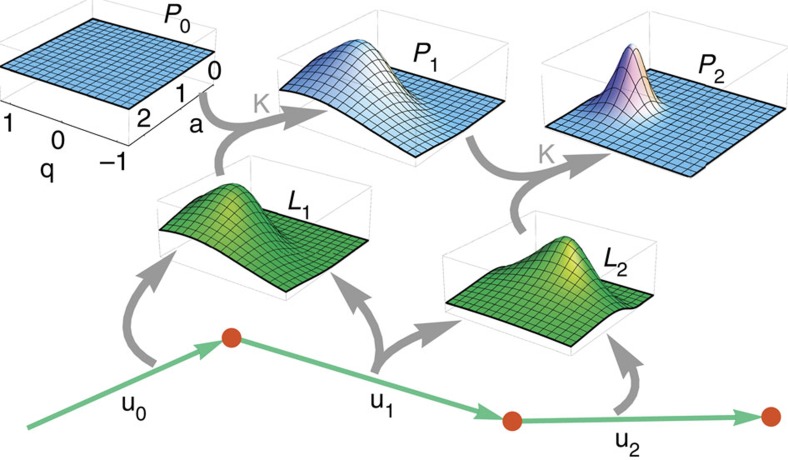
Bayesian inference of time-dependent random walk parameters. From two successive displacement vectors **u**_0_ and **u**_1_, the likelihood *L*_1_(*p*,*a*) (green) of the parameters can be computed. This distribution is multiplied (grey) with the prior guess *P*_0_(*p*,*a*) (blue). *K* denotes a transformation that accounts for temporal parameter evolution. This process is iterated in forward and backward (not shown) time direction, and the priors are combined.

To extract the time-dependent joint probability density *P*(*q*_*t*_,*a*_*t*_) of the parameters *q*_*t*_ and *a*_*t*_ from a sequence of displacements **u**_*t*_, we use sequential Bayesian updating. We start at time *t*=0 with a flat prior distribution *P*_0_(*q*,*a*) (see *P*_0_ in Fig. 2), which can be interpreted as a ‘first guess' about the parameter values. From the measured successive displacements **u**_0_ and **u**_1_, we compute the likelihood distribution *L*_1_(*q*,*a*) (see *L*_1_ in Fig. 2), which provides a first information about probable parameter values.

The prior distribution *P*_0_ and the likelihood distribution *L*_1_ are multiplied to obtain the posterior distribution *P*_0_*L*_1_, which updates our guess of the parameter values for the next time step. In the case of a temporally homogeneous process with constant parameters, iterative multiplication of the posterior distributions with the likelihood distributions, *P*_*t*_=*P*_*t*−1_*L*_*t*_ (Fig. 2), would yield an increasingly accurate estimate of the parameter values. For heterogeneous processes, however, the possibility of changing parameters has to be taken into account. This is achieved by a transformation *K* of the posterior distribution, *P*_*t*_=*K*(*P*_*t*−1_*L*_*t*_). The transformation *K* (blurring and preventing the posterior distribution to fall below a small cutoff value) is chosen such that both gradual and abrupt parameter changes can be identified (see Methods section). Finally, we perform the same sequential parameter inference in the reverse time direction (not shown in Fig. 2) and combine both distributions.

**Figure 3 f3:**
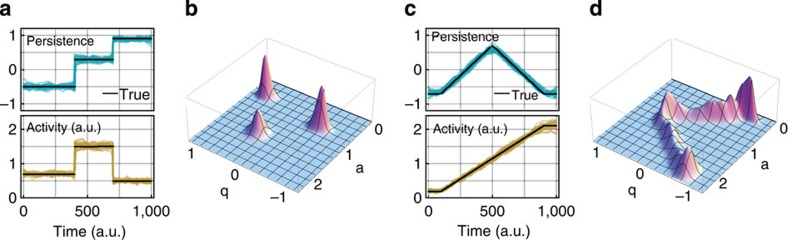
Validation of Bayesian parameter inference with simulated data. The Bayesian method can reliably extract both abrupt (**a**,**b**) and gradual (**c**,**d**) parameter changes. (**a**,**c**) The prescribed parameter evolution (black) and reconstructions of persistence (blue), and activity (yellow) from multiple simulations. (**b**,**d**) The time-averaged joint posterior distributions of the parameters.

We validate this method by simulating random walk trajectories from prescribed stepwise (Fig. 3a) or gradually (Fig. 3c) changing parameter sequences {(*q*_*t*_,*a*_*t*_)}. We then reconstruct the parameter sequences from the simulated trajectories by sequential Bayesian inference. The mean values of the posterior distributions fluctuate around the ‘true' parameter values, but follow the prescribed time evolution closely, both for abrupt (Fig. 3a) and gradual (Fig. 3c) parameter changes. We also find that the Bayesian method is superior to a maximum likelihood estimation with a sliding time window. The maximum likelihood estimation method cannot handle abrupt and gradual parameter changes equally well, and the user must find a compromise between long time windows that wash out sudden parameter jumps and short windows that lead to noisy results (Supplementary Note 4).

### Heterogeneity of measured random walks

**Figure 4 f4:**
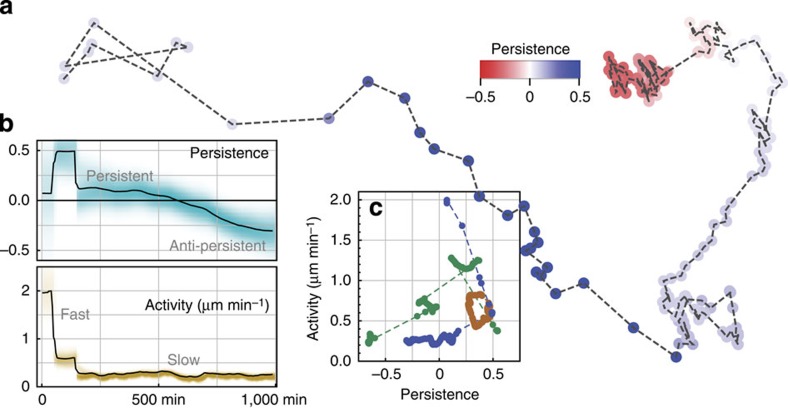
Temporal heterogeneity of MDA-MB-231 tumour cell migration on uncoated plastic. (**a**) Example cell trajectory, with colours representing the posterior mean of the momentary persistence *q*_*t*_ according to the colour bar. (**b**) Persistence (top) and activity (bottom) of the same cell as a function of time. Shading intensity is proportional to the probability density distribution. The cell starts in a highly active and persistent state, switches within 200 min to a more inactive but still persistent state and then gradually changes from persistent (blue parts of trajectory) to anti-persistent (red parts) behaviour during the following 800 min. (**c**) Activity versus persistence ((*q*,*a*)-plane) for three individual cells (green, brown and blue) of the same type, migrating on uncoated plastic. The blue cell is the same cell shown in **a** and **b**. The dashed lines connect subsequent sampling points.

We next apply the Bayesian inference method to measured cell trajectories. An example for the parameter evolution of a cell migrating on uncoated 2D plastic is shown in Fig. 4. We find large variations of cell behaviour, both with time (Fig. 4a,b) and between individual cells (Fig. 4c). By plotting the cell activity versus persistence for all time points, we further find that individual cells can occupy different regions of the (*q*,*a*) parameter plane (Fig. 4c). Some cells remain in a small compact region of the (*q*,*a*)-plane during the entire measurement period (brown), whereas others jump between disjunct subregions (green) or continuously change their parameters over time (Fig. 4c).

### Superstatistical data evaluation

**Figure 5:**
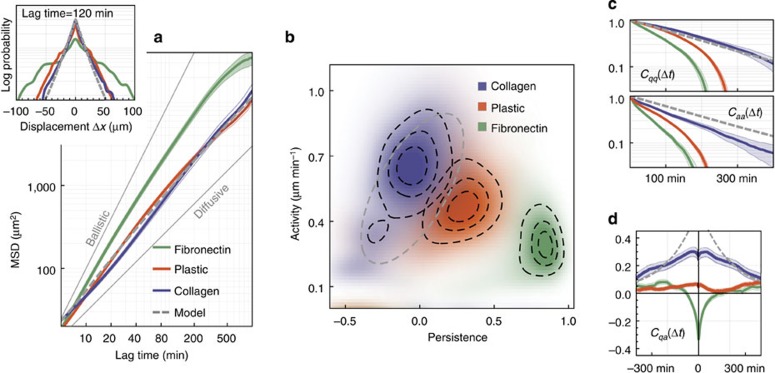
Statistical evaluation of cell migration data. Conventional (**a**) and superstatistical (**b**–**d**) evaluation of migration data, ensemble-averaged over all cells in the same environment. The MSDs (**a**, main) grow superdiffusively with lag time, according to power laws with exponents 1.3 in collagen (*n*=65 cells from five experiments) and on uncoated plastic (*n*=177 cells from eight experiments), and 1.7 on fibronectin-coated plastic (*n*=69 cells from three experiments). The thin lines around each MSD curve indicate the s.e.m. (obtained with the bootstrap method). The SWDs are close to exponential for a lag time of 120 min (**a**, inset), as well as for other measured lag times (Supplementary Note 3). MSD and SWD show no differences between migration in collagen and on uncoated plastic. By contrast, large differences between all three environments are seen in the time-averaged joint parameter distributions (**b**), and also in the auto-correlations (**c**), and cross-correlations (**d**) of the parameters. Dashed black lines in **b** represent the 10, 25 and 50% credible regions. Shading in **c** and **d** corresponds to 1 s.e.m.. Dashed grey lines in **a**–**d** correspond to the superstatistical model of migration in collagen gels.

*Joint probability distributions*. We average the posterior distributions *p*(*q*,*a*) for all time points and all cells measured in the same environment (Fig. 5b). In contrast to MSD and SWD, the ensemble-averaged posterior distributions show large differences between all three environments. The peak position of the distribution shows the lowest persistence and highest activity for collagen, and the highest persistence and lowest activity for fibronectin-coated plastic. Moreover, the spread of the distributions indicates that migration in collagen gels is more heterogeneous compared with migration on plastic. The *p*(*q*,*a*) distributions thus provide characteristic ‘fingerprints' of the migration environments that can be used for automatic trajectory classification. In a ‘leave-one-out' cross-validation, we were able to assign ∼90% of the cell trajectories to the correct environment (see Methods section).

*Parameter correlations*. The auto- and cross-correlations of the time-dependent parameters *q*_*t*_ and *a*_*t*_ reveal even larger differences between migration strategies in 2D versus 3D environments. Auto-correlation times are noticeably longer in a 3D environment (Fig. 5c), where the local biopolymer fibre configuration provides a guiding or trapping microstructure that influences a given migration mode for long time periods. Large differences between different environments are also visible in the cross-correlations of the time-dependent parameters (Fig. 5d). On fibronectin-coated plastic, persistence and activity are negatively correlated for up to 100 min. This is consistent with the long-known observation that on highly adhesive surfaces, cells maintain persistent motion by performing sequences of small steps along the same direction^25^. The continuous gliding motion is not seen on less adhesive, uncoated plastic surfaces. Instead, we observe a weakly positive cross-correlations between *q*_*t*_ and *a*_*t*_. In collagen, we find strong positive correlations between *q*_*t*_ and *a*_*t*_, consistent with the observation that cells intermittently cover large distances with high directional persistence guided by long protrusions (Supplementary Movies 1 and 2).

Note that the activity parameter *a*_*t*_ should not be interpreted literally as the momentary cell speed *u*_*t*_, but as a scale parameter that—together with *q*_*t*_—determines the most probable value of the cell speed. To clarify this point, we also investigate the correlation between persistence *q*_*t*_ and momentary cell speed *u*_*t*_. For migration on coated and uncoated plastic surfaces, we find a positive correlation between *q*_*t*_ and *u*_*t*_ (Supplementary Note 6). A similar relationship has been reported for a variety of different cell types migrating on fibronectin-coated surfaces^26^. In collagen, however, persistence and cell migration speed are uncorrelated (Supplementary Note 6).

### Effect of local microenvironment

**Figure 6:**
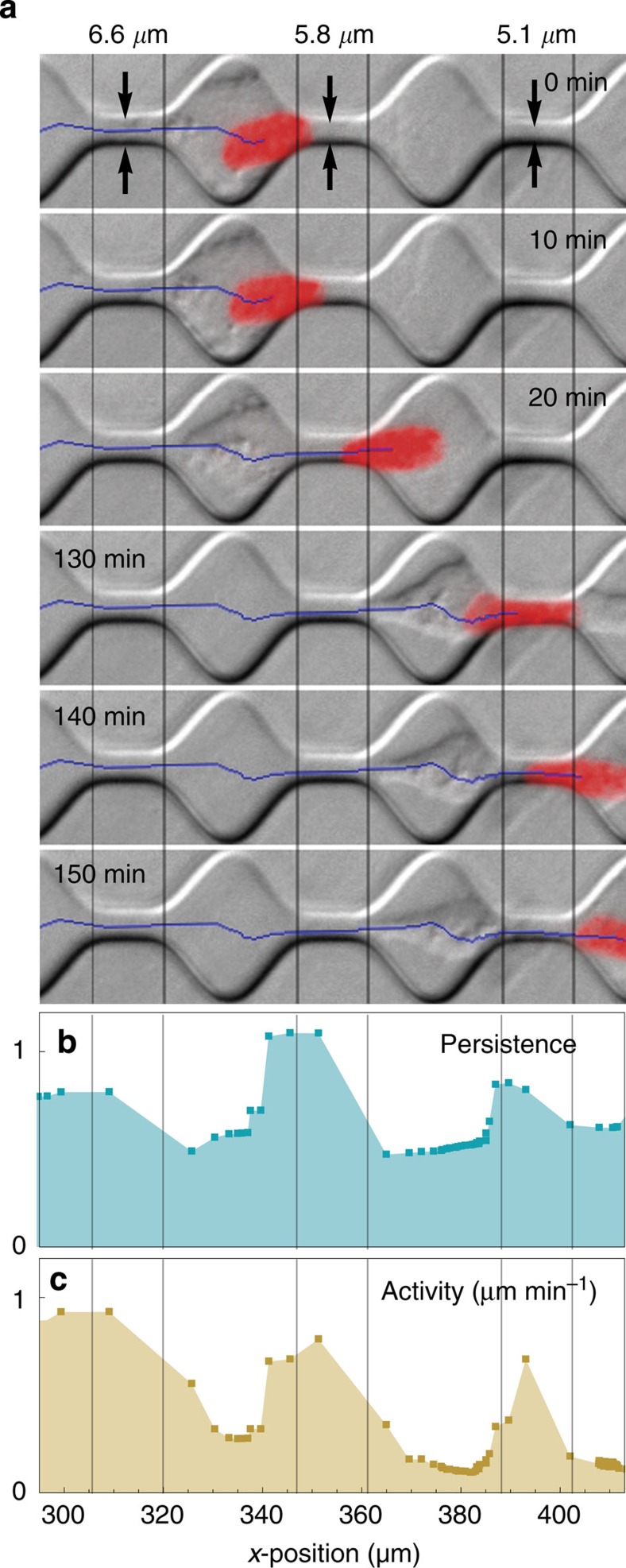
Cell in a microstructured channel array. (**a**) Primary breast cancer cell migrating through a linear array of sequentially narrowing channels and wider chambers. The cell nucleus (red) is continuously tracked, with the centroid positions marked in blue. Persistence (**b**) and activity (**c**) are high when the nucleus transverses narrow channels, and decrease when the nucleus enters the wider chambers.

In the previous section, we have tacitly assumed that the local microenvironment has an immediate effect on migration persistence and activity. To test this assumption, we use a microstructured environment and measure cell migration through a linear (1D) array of sequentially narrowing channels and wider chambers. After extracting the time-dependent parameters *q*_*t*_ and *a*_*t*_ from individual cell trajectories (Fig. 6a), we plot *q*_*x*_ (Fig. 6b) and *a*_*x*_ (Fig. 6c) versus the *x*-position.

The precise migration mechanism of different cell types through such environments is not well understood and may involve integrin-mediated adhesion-dependent^27^ or adhesion-independent^28^ strategies. Regardless of the migration mechanism, our microstructured environment forces the cells to adapt to different degrees of confinement in rapid succession. A cell that enters a channel first has to polarize and deform its nucleus. It can then transit the channel with high persistence and activity. When the cell nucleus exits the narrow channel and enters the wider chamber, persistence and activity decrease markedly. Thus, the superstatistical migration parameters are strongly correlated with the local properties of the environment.

### Superstatistical modelling

We construct a series of simple models of cell migration that approximate the statistical properties of *q*_*t*_ and *a*_*t*_ found in the data. All models are based on an AR-1 process. The superstatistical parameters *q*_*t*_ and *a*_*t*_ switch to new values, drawn from fixed distribution *p*_model_(*q*,*a*), after exponentially distributed time intervals with mean value *T*_model_. This regime-switching approach leads to exponentially decaying auto-correlations of the parameters with correlation time *T*_model_. We choose *T*_model_=200 min taken from migration experiments in collagen (Fig. 5c). The parameter distribution *p*_model_(*q*,*a*) is modelled as a bivariate Gaussian, centred at the main peak of the experimentally observed distribution *p*(*q*,*a*) (Fig. 5b).

We first consider the limit of zero variance for *p*(*q*,*a*), which corresponds to a homogeneous correlated random walk with constant *q* and *a*. In this case, the MSD is crossing over from a ballistic (slope 2) to a diffusive (slope 1) behaviour at a specific lag time that depends only on the persistence *q*. Increasing the variance of *q* generates a continuous mixture of crossover times, and the MSD starts to resemble a power law (Supplementary Note 1). In addition, the SWD becomes leptocurtic, but it does not show the exponential distribution found in the experiments. Finally, using an asymmetric bivariate normal distribution with positive correlations between *q* and *a* (Fig. 5b, dashed grey ellipse), the SWD, MSD and correlation functions match the measured data nearly perfectly (Fig. 5a,c,d, dashed grey line).

This example demonstrates how superstatistics can recapitulate the anomalous features of heterogeneous random walks by mapping the complexity of the system into a suitable distribution of parameter values *p*_model_(*q*,*a*), while keeping the underlying stochastic process simple.

## Discussion

In this study, we have applied the superstatistical framework to the specific example of tumour cell migration in environments with different dimensionality. The same approach, including the particular choice of the AR-1 process as a local model, can be used for many other heterogeneous random walks in life sciences. For this purpose, we provide a Python implementation of the Bayesian algorithm for inferring the time-dependent parameters *q*_*t*_ and *a*_*t*_ from random walk trajectories (Supplementary Software 1).

In principle, a sequential, grid-based inference of superstatistical parameters can also be performed by a Markov Chain Monte Carlo approach. In this case, the vector of model parameters to be inferred consists of the full set {(*q*_*t*_,*a*_*t*_)} of superstatistical parameters for all time points. In the past, Markov Chain Monte Carlo methods, mostly based on the Metropolis Hastings algorithm, exhibited serious convergence problems when applied to such high-dimensional parameter spaces. Only recently, a novel sampling method based on Hamiltonian Monte Carlo has markedly improved the convergence^29^. Our preliminary tests demonstrate that this new sampling algorithm can indeed find the parameter vector of a hierarchical superstatistical model, however, with a considerably longer computation time.

Our superstatistical framework can be readily adapted to more complex types of stochastic systems. In particular, the AR-1 process can be replaced by any parameterized model with a defined likelihood function. For example, fluorescent beads attached to the cytoskeleton of living cells show fluctuations that can be described by a particle diffusing in a harmonic potential well^30^,^31^. Due to cytoskeletal remodelling, the centre position of the potential well is changing on longer timescales. Together, this process can be modelled with an inhomogeneous random walk of the centre position, superposed with a harmonic overdamped oscillator^32^. As a final example, recordings of neural spike trains are frequently modelled as inhomogeneous Poisson processes with a time-dependent spike rate. In this case, sequential Bayesian inference can be used to extract the local spike rates from the time series of measured interspike intervals.

## Methods

### Cell culture and migration measurements

For migration experiments in collagen, on plastic and on fibronectin-coated plastic, we use MDA-MB-231 breast carcinoma cells (obtained from the American Type Culture Collection (ATCC)). Cells are cultured in 75 cm^2^ flasks in Dulbecco s modified Eagle's medium (DMEM) (1 g l^−1^ D-glucose) and 10% fetal bovine serum, 1% penicillin/streptomycin at 37 °C, 5% CO_2_ and 95% humidity. Cells are passaged every second day. Trypsin-ethylenediaminetetraacetic acid (Trypsin-EDTA) is used to detach cells.

To study cell migration on planar surfaces, we use tissue culture-treated plastic dishes with and without fibronectin coating (69 and 177 cells, respectively). In all 2D experiments, the sample time interval between frames was *δt*_2D_=1 min.

For 3D experiments, we use reconstituted collagen gels (Fig. 1a) with controlled material properties as a substitute for biological tissue. At a collagen concentration of 2.4 mg ml^−1^, these gels have an average pore radius of 1.3 μm and a shear modulus of 108 Pa (ref. 33). Cells are mixed with collagen solution before polymerization at a concentration of 15,000 cells per ml. The *x-*, *y-* and *z*-position of the cells within the collagen gel is determined from a characteristic intensity profile of the refraction pattern around the nucleus of the cell (inset of Fig. 1b). A 3D deconvolution of the intensity profile then defines the cell position with an accuracy of 2 μm (r.m.s.). Cell tracking is performed automatically in real time, and the cell position is used to keep the motorized microscope *x*–*y*-centred and *z*-focused onto the cell at all times. Using a time-sharing mode, we are able to observe and follow up to 20 individual migrating cells within the same cell culture well over prolonged time periods (24 h). We record discrete cell positions with a sample time interval of *δt*_3D_=2.5 min (Fig. 1c). Cells undergoing cell division during the time of observation were excluded. The number of analysed cells in collagen was 65.

We also study the migration of primary inflammatory ductal breast cancer cells (gift from Pamela Strissel and Reiner Strick, Womens Hospital, University Clinics Erlangen) within a microfabricated channel structure made of polydimethylsiloxan. The structure has a constant height of 3.7 μm and consists of 15 consecutive channels with diameters decreasing from 11 to 1.7 μm, separated by 20 × 20-μm-wide chambers (Fig. 6a). After staining the cell nuclei with Hoechst 33342 (1 μg ml^−1^), the centre positions are tracked with a sample time interval of *δt*_1D_=5 min. For superstatistical evaluation, a cell is chosen that passed through two successive channels within 150 min.

### Bayesian parameter inference

Since the iterative updating of the parameter distribution described in this work is not analytically tractable, the presented algorithm is implemented using discretized probability distributions. Based on equally spaced parameter values *q*_*i*_ and *a*_*j*_ (*i*∈{1,2,..,*N*_*q*_}, *j*∈{1,2,..,*N*_*a*_}), a distribution *p*(*q*,*a*) can be approximated by a *N*_*q*_ × *N*_*a*_-dimensional matrix: (*p*(*q*,*a*))_*ij*_=*p*(*q*=*q*_*i*_,*a*=*a*_*j*_). The multiplication of two distributions is thus reduced to the element-wise multiplication of two matrices.

The prior distribution *P*_*t*_=*p*(*q*_*t*+1_,*a*_*t*+1_) holds the preliminary belief about the latent parameter values for the next time step, before seeing the corresponding data point. Using the data point **u**_*t*+1_, we subsequently update the prior distribution by multiplying it with the likelihood *L*_*t*+1_=*p*(**u**_*t*+1_|*q*_*t*+1_,*a*_*t*+1_;**u**_*t*_) that describes the probability of observing a certain measurement **u**_*t*+1_, given the values of the latent parameters (and the previous measurement **u**_*t*_).

For the underlying AR-1 process, the likelihood is given by


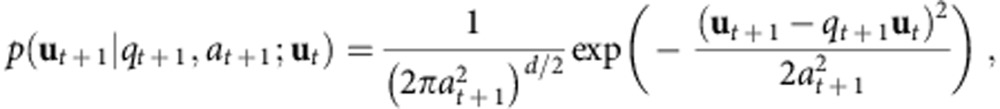


where *d* states the number of dimension of the velocity vectors (two in this study). Note that the inference method can also be applied to other underlying stochastic processes with more complicated likelihood functions. As our approach uses only the numerical values of the likelihood for discrete points of the (*q*_*t*_,*a*_*t*_)-grid, the likelihood need not be expressed analytically as long as it can be computed numerically.

The next prior *P*_*t*+1_ is computed from the posterior distribution *P*_*t*+1_=*K*(*P*_*t*_*L*_*t*+1_), with *K* being a transformation that accounts for both gradual and abrupt parameter changes as follows: To allow for abrupt parameter changes, we set the minimal probability of the posterior distribution to *p*_min_=10^−7^


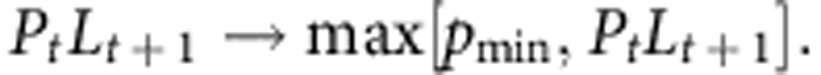


To allow for gradual parameter changes, we blur the distribution by convolution with a box kernel *B* of radius *R*=0.03 defined as


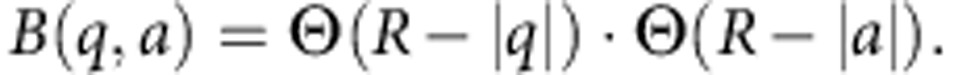


Here, Θ(*x*) is the Heaviside step function. The posterior distribution of the parameters is normalized at every time step, since the transformation *K* does not preserve normalization. A systematic procedure to find optimal values for the two parameters *p*_min_ and *R* is given in the Supplementary Note 5.

Starting with a flat prior *P*_0_ and moving forward in time using the iteration described above, a series of ‘forward' priors 
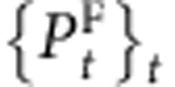
 is generated. In the same way, we can start the iteration at the end of a trajectory, and build a series of ‘backward' prior distributions 
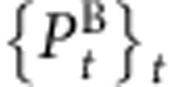
. Finally, for each time step *t*, we multiply the *t*−1 and *t*+1 priors with the likelihood *L*_*t*_ to compute the final posterior distribution of the parameters (*q*_*t*_,*a*_*t*_), so that 
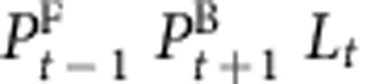
. Note that the inference algorithm is run in both directions of time to ensure that for each estimated parameter pair (*q*_*t*_,*a*_*t*_), all measured data points are taken into account and not only those of earlier times 0…*t*. In principle, however, the algorithm can also be used only in the forward direction, which may be useful for online analysis of a data stream.

### Temporal and ensemble averages

Throughout this paper, the symbol 〈*f*_*t*_〉_*t*_ denotes temporal averaging over all discrete time points. For our data evaluation (SWD, MSD and auto- and cross-correlations), we have additionally ensemble-averaged the time-averaged properties over the individual cells of the same migration environment.

### Auto- and cross-correlations

The auto-correlation *C*_*qq*_(Δ*t*) of the persistence parameter *q*_*t*_ is defined in the standard way as 
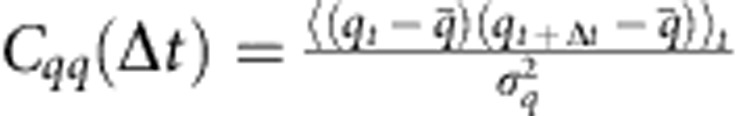
, where 
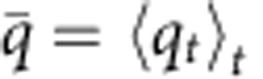
 is the temporal average and 
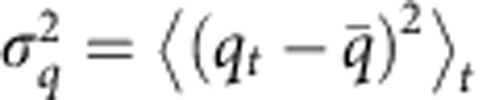
 is the variance of the parameter. The definition of the activity auto-correlation *C*_*aa*_(Δ*t*) is analogous. Finally, the cross-correlation *C*_*qa*_(Δ*t*) between the two parameters is defined as 
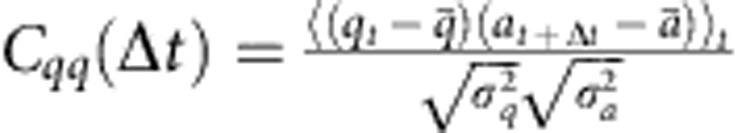
.

### Superstatistical modelling of cell migration

To model the statistical properties of cell trajectories in collagen (Fig. 5, grey dashed lines), we use a superstatistical regime-switching process with an average switching time of *τ*=200 min. Parameter values (*q*_*t*_,*a*_*t*_) are drawn from a bivariate Gaussian distribution, (*q*_*t*_,*a*_*t*_)∼*N*(*μ*,Σ), centred around the mean ***μ***=(*μ*_*q*_,*μ*_*a*_)=(−0.05,0.55). The covariance matrix is 
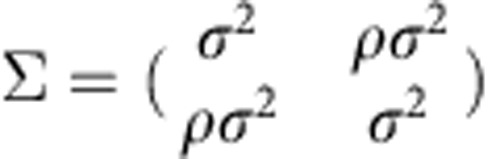
 with *σ*=0.3 and *ρ*=0.65. The 50% credibility region of the distribution is shown in Fig. 5b as a grey dashed ellipse. The values of *q*_*t*_ are restricted to the interval [−1,1].

### Environment-specific cell classification

For ‘leave-one-out' cross-validation, we calculate the squared deviation *D* between the time-averaged posterior distribution of a single cell, denoted *p*_single_(*q*,*a*), and each of the three ensemble- and time-averaged distributions *p*_env_(*q*,*a*) (excluding that one cell). The calculation of the squared deviation is carried out as a sum over the *N*_*q*_ × *N*_*a*_-grid:


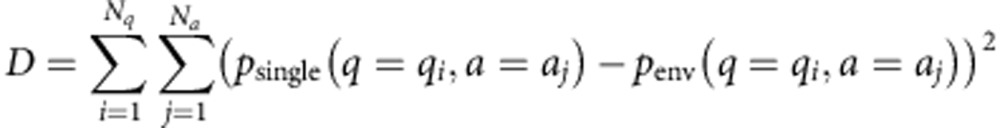


A cell is counted as correctly classified if the deviation to its true environment is the smallest, compared with the other two environments.

## Additional information

**How to cite this article:** Metzner, C. *et al.* Superstatistical analysis and modelling of heterogeneous random walks. *Nat. Commun.* 6:7516 doi: 10.1038/ncomms8516 (2015).

## Supplementary Material

Supplementary Information Supplementary Figures 1-12 and Supplementary Notes 1-6

Supplementary Movie 1 Time-lapse phase contrast images of an MDA-MB-231 tumour cell migrating within a collagen gel over the time course of 7 h. Time is indicated in the upper-left corner (in h and min). The microscope is automatically following the movement of the cell to keep it in the focus position at all times. Note that the cell centre moves persistently despite frequent changes in cell shape.

Supplementary Movie 2 Time-lapse integrated modulation contrast images of MDA-MB-231 tumour cells migrating within a collagen gel over 250 min. Time is indicated in the lower right corner in min. The cell probes its environment with a long protrusion that guides the movement of the cell body.

Supplementary Movie 3 Time-lapse integrated modulation contrast images of MDA-MB-231 tumour cells migrating on tissue treated plastic over 18 h. Time is indicated in the upper left corner (in h and min). Cells are retracing their own paths or that of other cells. The scale bar is 100 μm.

Supplementary Movie 4 Time-lapse images of MDA-MB-231 tumour cells migrating on fibronectin-coated plastic over 14 h. Time is indicated in the upper-left corner in h and min. Note that cells are moving very persistently, only rarely retracing their paths. The scale bar is 100 μm.

Supplementary Software 1 Python scripts and a corresponding readme-file. The first script, 'simulatedExamples.py', is a demonstration of our Sequential Bayesian Inference algorithm based on simulated data and provides extended documentation of the implementation details. The second script, 'dataAnalyzer.py', allows users to analyze arbitrary two-dimensional velocity data using Sequential Bayesian Inference. The readme-file provides an instruction to the requirements and usage of the software.
