# Supplementary material for: Superstatistical analysis and modelling of heterogeneous random walks
Source: Nat Commun. 2015 Jun 25;6:7516. doi: 10.1038/ncomms8516 (PMC4491834; doi:10.1038/ncomms8516)
Supplement: Supplementary Information — Supplementary Figures 1-12 and Supplementary Notes 1-6 [file ncomms8516-s1.pdf]

Supplementary Note 1

Emergence of anomalous features in time-averaging statistics of heterogeneous random walks

The traditional tools for analyzing random walks are step width distributions (SWD) and mean squared displacements (MSD). For a given trajectory time series $\left\{\vec{R}_t^{(s)}\right\} = \left\{(x_t^{(s)}, y_t^{(s)}, z_t^{(s)})\right\}$, these statistical measures are computed by averaging certain quantities of interest over the time index t. An additional average may be performed over an ensemble of different measured time series s. In particular, the SWD in x-direction is defined as

$$P(\Delta x, \Delta t) = \left\langle \delta\left(\Delta x - (x_{t+\Delta t}^{(s)} - x_t^{(s)})\right)\right\rangle_{t,s} \quad (1)$$

and the MSD is defined as

$$\overline{R^2}(\Delta t) = \left\langle |\vec{R}_{t+\Delta t}^{(s)} - \vec{R}_t^{(s)}|^2 \right\rangle_{t,s}. \quad (2)$$

In the case of an uncorrelated random walk, the SWD is a Gaussian distribution with respect to Δx, with a variance that increases linearly as a function of the lag-time Δt. The MSD also increases linearly for all lag-times.

For a homogeneous correlated random walk with correlation time τ, the behavior of the SWD and MSD depend on the lag-time Δt. In the regime of short lag-times, $\Delta t \ll \tau$, the SWD is non-Gaussian and the MSD grows quadratically (ballistic motion). In the regime of long lag-times, $\Delta t \gg \tau$, the SWD is Gaussian and the MSD grows linearly (diffusive motion), just as in an uncorrelated random walk. The correlation time τ thus defines the cross-over time between the slope 2 and slope 1 in a double-logarithmic plot of the MSD versus lag-time (colored lines in Supplementary Figure 1A).

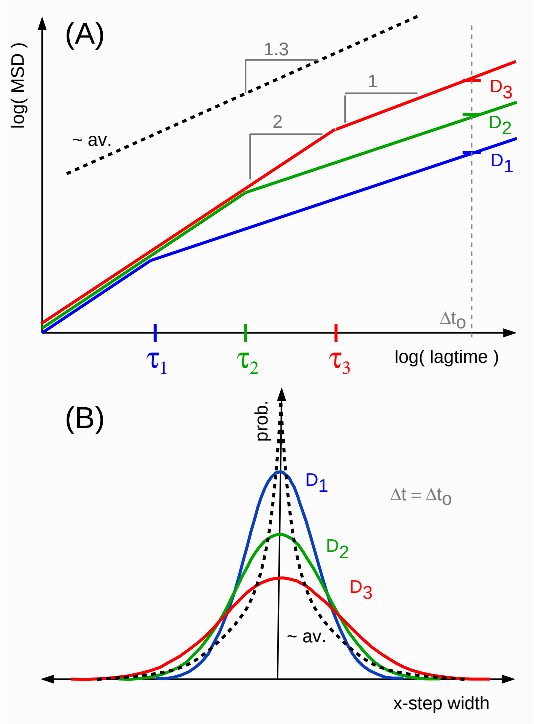

Supplementary Figure 1: ***Emergence of anomalous mean squared displacements and step width distributions****. Anomalous statictics can be due to superstatistical fluctuations of the velocity decorrelation time τ. (A): Mean squared displacements (MSD, colored lines) for three different decorrelation times τ_s, which mark the cross-over points from the ballistic regime to the diffusive regime. Averaging over an ensemble with suitably distributed τ_s can result in a power law with fractional exponent (black dashed line). For lag-times $\Delta t \gg \tau_s$, each case s can be described by an effective diffusivity D_s. (B): Step width distributions (SWD, colored lines) at a sufficiently long lag-time Δt_0 are Gaussians with a variance proportional to D_s. Averaging over an ensemble of narrow and wide normal distributions results in a leptocurtic distribution (black dashed line).*

Next, we consider a heterogeneous ensemble $\left\{\vec{R}_t^{(s)}\right\}$ of temporally homogeneous correlated random walks, each with a different correlation time τ_s. In this case, the SWD in the regime of long lag-times is an average over Gaussians of different variance (colored curves in Supplementary Figure 1B), which results in a leptocurtic and therefore anomalous distribution (black dashed curve in Supplementary Figure 1B). Similarly, for a sufficiently wide and dense distribution of τ_s, the individual cross-over times between ballistic (slope 2) and diffusive (slope 1) regimes average out, and the MSD assumes an anomalous shape. In particular, it can be shown that heterogeneity of this kind can lead to an almost exponential SWD and a MSD that resembles a power law with a fractional exponent (dashed line in Supplementary Figure 1A). For an analytical derivation, see arXiv:1207.2242).

Finally, note that a temporally heterogeneous random walk in which the correlation time τ varies slowly as a function of time t has a similar effect as the heterogeneous ensemble above. This demonstrates that anomalous features emerge naturally in the SWD and MSD if these statistics are applied to heterogeneous random walks.

Supplementary Note 2

Random walk models in the superstatistical framework

In a superstatistical framework, random time series are described, on the lowest hierarchical level, by a simple stochastic 'base model' with a small set of parameters. In this paper, we have chosen the AR-1 process with a persistence parameter q and an activity parameter a. Keeping these superstatistical model parameters constant corresponds to a temporally homogeneous random process. For example, the AR-1 process with constant parameters q and a corresponds to a homogeneous persistent random walk (Supplementary Figure 2).

In order to describe more complex time series, in particular measured random walks that show obvious temporal heterogeneity, it is possible to resort to higher order base models, such as the class of AR-n processes with $n > 1$. However, this approach is misguided in cases where the temporal heterogeneity is caused by time- or position-dependent external influences that cannot be easily incorporated into the base model. A standard example of a random walk that becomes heterogeneous due to external effects is a particle diffusing in a medium with different local temperatures.

The superstatistical framework acknowledges external time-dependent effects and models them directly as a higher level stochastic process that affects the parameters of the base model. In our case, the two parameters q_t and a_t of the AR-1 process are allowed to change at any time step, according to some higher order process. By this way, heterogeneous random walks of arbitrary complexity can be described by a hierarchy of low-order stochastic models. A simple example that highlights externally controlled heterogeneity is the intermittent random walk, in which a particle switches randomly between phases of ballistic and diffusive motion. This heterogeneous process can be described by a two-level superstatistical model with an AR-1 process as the base model and a Markov switching process as the second level model (Supplementary Figure 3).

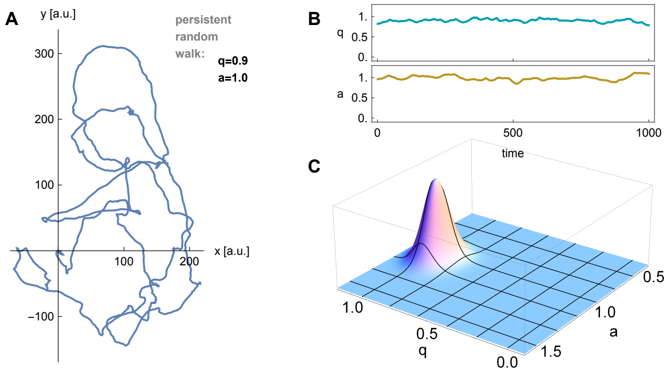

Supplementary Figure 2: ***Persistent random walk in 2D***. *Simulated random walk, generated by using an AR-1 process with constant values* $q = 0.9$ *for the persistence and* $a = 1$ *for the activity. (A): Sample trajectory. (B): Superstatistical parameters* q_t *and* a_t *as a function of time, reconstructed from the sample trajectory. (C): Corresponding time-averaged posterior distribution* $p(q, a)$. *The single peak indicates that the random walk is temporally homogeneous.*

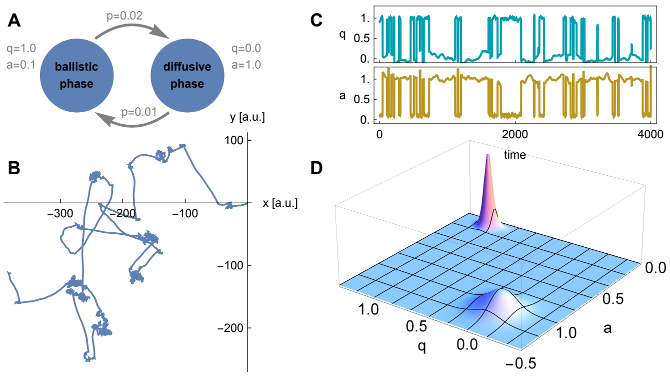

Supplementary Figure 3: ***Intermittent random walk in 2D***. *Simulated random walk, generated by an AR-1 process in which the superstatistical parameters switch stochastically between a ballistic phase* $(q = 1, a = 0.1)$ *and a diffusive phase* $(q = 0, a = 1.0)$. *(A) Representation of the Markov process that controls the stochastic switching between the two phases. (B) Sample trajectory. (C): Superstatistical parameters* q_t *and* a_t *as a function of time, reconstructed from the sample trajectory. (D): Corresponding time-averaged posterior distribution* $p(q, a)$. *The two peaks directly reflect the two distinct phases in this temporally heterogeneous random walk.*

Supplementary Note 3

Step width distributions

In the main part of the paper, we have argued that the traditional, globally averaging statistical measures, such as the mean squared displacements (MSD) and the step width distributions (SWD), are often masking important differences between distinct types of random walks. In particular, we have shown that the SWD of MDA-MB-231 breast carcinoma cells migrating in a collagen network and on a plastic dish are practically indistinguishable on a time scale of 120 min. Here, we demonstrate that the SWDs in the two environments are also nearly identical for all lag times between 5 min and 500 min (Supplementary Figure 4, blue and red lines). The form of the distributions remains approximately exponential for the whole range of lag-times. In contrast, the SWD for migration on fibronectin coated surfaces (green lines) is significantly different from the other two environments for all lag times larger than 10 min.

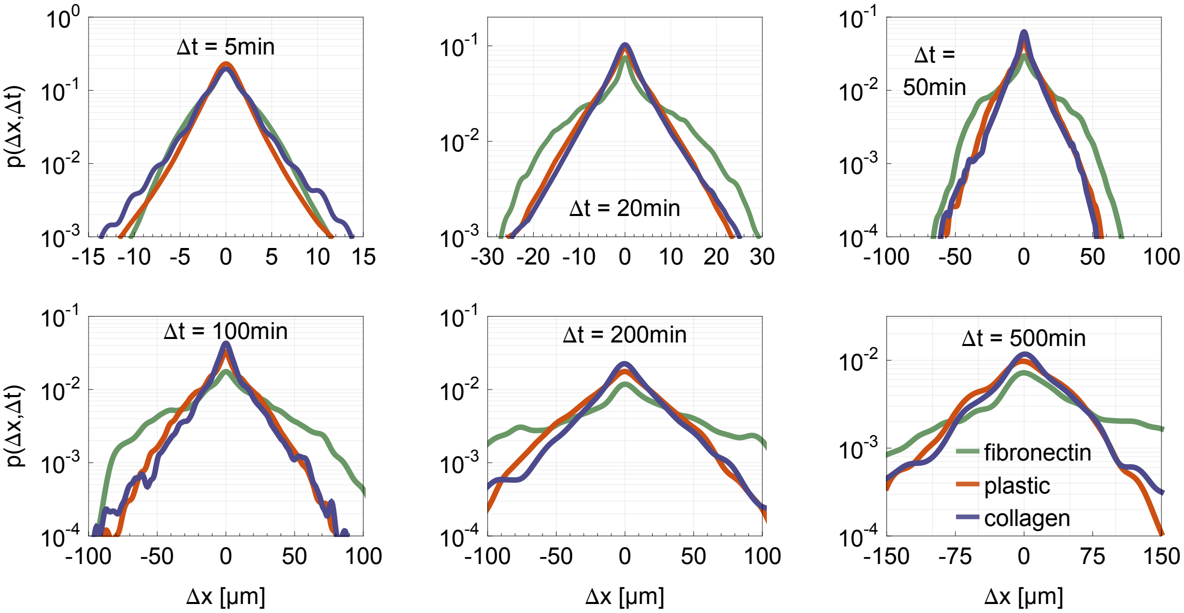

Supplementary Figure 4: ***Step width distributions (SWDs)****. Shown are SWDs (for six different lag-times) of MDA-MB-231 breast carcinoma cells in a collagen network (blue), on uncoated plastic (red) and on plastic coated with the adhesive ligand fibronectin. The distributions in collagen and on plastic are similar for all lag-times, although the migration mechanism of the cells is drastically different in these two environments.*

Supplementary Note 4

Comparison of Bayesian parameter inference with sliding window-based Maximum Likelihood estimation

In the main text, we have shown that the presented Bayesian method yields accurate results for simulated random-walk trajectories with abruptly switching (Fig. 3a,b) as well as linearly changing parameter values (Fig. 3c,d). Here, we provide an additional test case by combining both abrupt and gradual, sinusoidal parameter changes (Supplementary Figure 5). Based on these three test cases (regime-switching, linear, and sinusoidal), we compare our Bayesian method to an alternative inference method for heterogeneous random walks, namely the Maximum Likelihood estimation using a sliding window.

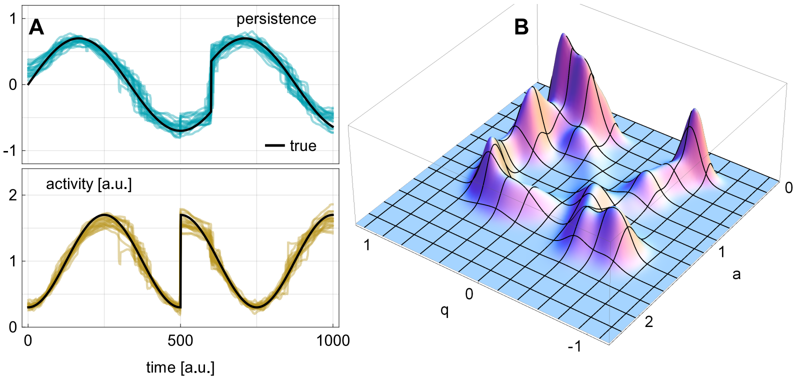

Supplementary Figure 5: ***Evaluation of the Bayesian inference method****. We use simulated trajectories with abrupt as well as gradual, sinusoidal parameter variations. (A) The reconstructed posterior mean values of persistence and activity from 20 simulated trajectories (color) are plotted together with the true values (black). (B) The time-averaged posterior distribution corresponding to a single trajectory reveals the complex correlations between the two parameters, persistence and activity.*

Sliding window analysis

The standard method for inferring time-dependent parameter changes in time series is the sliding window method, whereby the time series is subdivided into overlapping segments. For each segment, the parameters (activity and persistence in our case) are assumed to be constant and can be estimated using a maximum likelihood approach. Considering a homogeneous AR(1)-process for times t' within the interval $I_t = \{t - w/2, ..., t + w/2\}$ of length w, centered around some point in time t, with persistence q_t and activity a_t, one can state the log-likelihood for these parameters as follows:

$$\log\ p\left(\{\vec{u}_{t'}\}_{t' \in I_t} | q_t, a_t\right) \ \propto \sum_{t' \in I_t} \left(-\frac{\left(\vec{u}_{t'} - q_t \vec{u}_{t'-1}\right)^2}{2a_t^2} - \log\left(2\pi a_t^2\right) \right) .$$

Here, we consider a two-dimensional trajectory. Maximizing the log-likelihood with respect to q_t and a_t yields the following Maximum Likelihood estimators:

$$\hat{q}_t = \frac{\sum_{t' \in I_t} \quad \vec{u}_{t'} . \vec{u}_{t'-1}}{\sum_{t' \in I_t} \quad \vec{u}_{t'-1} . \vec{u}_{t'-1}} \quad \text{and} \quad \hat{a}_t = \sqrt{\frac{1}{2w} \sum_{t' \in I_t} \left(\vec{u}_{t'} - \hat{q}_t \vec{u}_{t'-1}\right)^2} \ ,$$

where (.) denotes the dot product. Note that the estimator of a_t depends on the estimated value of q_t. To analyze a heterogeneous time series using these estimators, the time series is partitioned into overlapping segments of length w – thus the term 'sliding window'. This segmentation allows us to estimate the local persistence and activity over the entire trajectory.

Method comparison

We use the mean squared error (mse) of the estimated parameter sequences with respect to the true parameter sequences to assess the quality of the two methods:

$$\text{mse}\left(\{(q_t, a_t), (\hat{q}_t, \hat{a}_t)\}\right) = \frac{1}{2N'} \sum_t \left((q_t - \hat{q}_t)^2 + (a_t - \hat{a}_t)^2 \right) \ ,$$

where N' denotes the length of the estimated parameter sequence. The ratio of the errors between both methods allows for a direct comparison:

$$r(w) = \frac{\text{mse}\left(\{(q_t, a_t), (\hat{q}_t, \hat{a}_t)_{\text{Bayes}}\}\right)}{\text{mse}\left(\{(q_t, a_t), (\hat{q}_t, \hat{a}_t)_{\text{ML}(w)}\}\right)} \ .$$

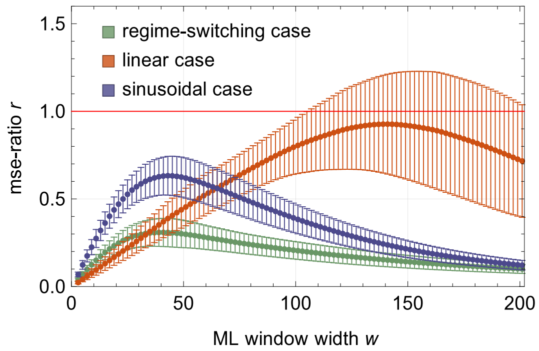

Supplementary Figure 6: ***Comparison of Bayesian inference with the Maximum Likelihood (ML) approach.*** *Based on the mean squared error ratio, the Bayesian method is directly compared to the ML approach for a variety of window sizes. With an average mse ratio smaller than one for all three test cases, the Bayesian method is found to be superior, regardless of the window size for the ML approach.*

We estimate r for different window widths ranging from 3 to 200 data points. For all three test cases (regime-switching, linear, and sinusoidal), and regardless of the chosen width of the sliding window, the Bayesian algorithm attains a smaller error on average ($r < 1$, see Supplementary Figure 6). Apart from the smaller estimation error, the Bayesian approach provides an advantage especially for small data sets. For the sliding window analysis, no parameters can be estimated during a time period of $w/2$ at the beginning and the end of the time series, due to the finite width of the sliding window. By contrast, the Bayesian method yields $N - 1$ estimates for N data points. Furthermore, the assumption of constant parameters q and a within each window of length w is an inherent limitation of the sliding window approach, since it is violated for most heterogeneous time series. Finally, the choice of the window size w strongly affects the resulting reconstruction of the time-varying parameters. For small window sizes, the Maximum Likelihood method can detect sudden parameter changes but shows large, erroneous fluctuations in the estimated parameter sequence (Supplementary Figure 7, green line). With increasing window size, these erroneous fluctuations diminish, but sudden parameter changes become increasingly blurred (Supplementary Figure 7, yellow and red).

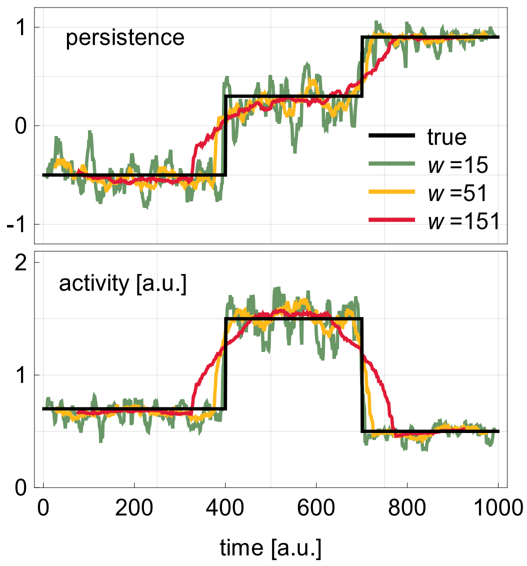

Supplementary Figure 7: ***Influence of window size***. *Effect of window size on Maximum Likelihood estimation in a regime-switching case (see Fig. 3). The true parameter values (black) are shown together with the estimated persistence and activity for different window sizes (color).*

Supplementary Note 5

Choice of the kernel parameters p_{min} and R.

In the main text, we have presented a sequential Bayesian method to reconstruct the superstatistical parameters q_t and a_t from a measured random walk time series. In each time step, the posterior distribution $P_t(q, a)$ is given a minimum probability $p_{min} = 10^{-7}$, which accounts for the possibility of abrupt parameter jumps to any value within the grid. Furthermore, the posterior distribution is convolved with a box kernel of radius $R = 0.03$, which assigns small (gradual) parameter changes a larger probability compared to large jumps.

In general, the two control parameters p_{min} and R do not require a precise fine tuning, but setting them outside a certain viable range leads to predictable problems: In general, too small values for the control parameters lead to a blurring of rapid parameter changes, whereas too large values lead to overfitting. We illustrate the effects of wrongly chosen control parameters p_{min} and R in Supplementary Figures 8 and 9.

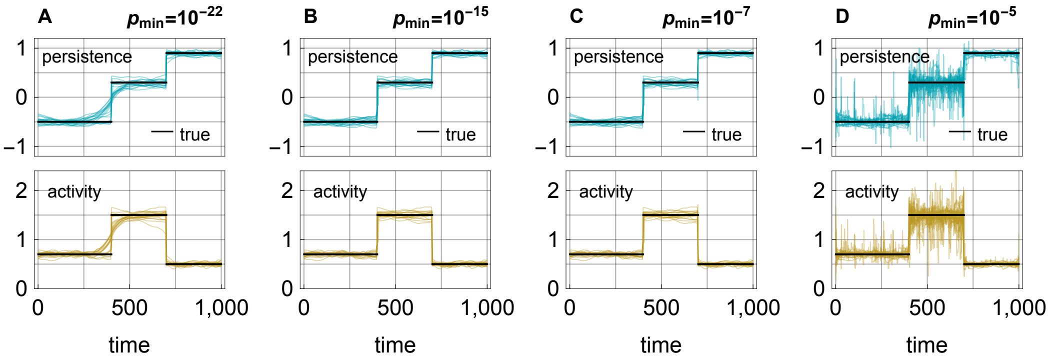

Supplementary Figure 8: ***Effect of kernel parameters for sudden changes of persistence and activity.*** *Effects of a too small (A), about optimal (B,C) and too large (D) setting of the control parameter* p_{min} *on the reconstruction of abruptly jumping superstatistical parameters* q_t *(top) and* a_t *(bottom). Note that the reconstruction quality degrades only very weakly, even if the control parameter deviates from the optimum value by orders of magnitude.*

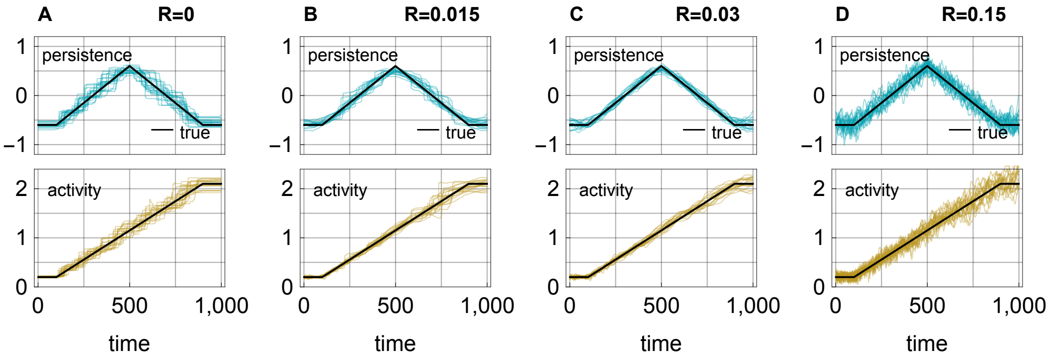

Supplementary Figure 9: ***Effect of kernel parameters for gradual changes of persistence and activity.*** *Effects of a too small (A), about optimal (B,C) and too large (D) setting of the control parameter* R *on the reconstruction of more gradually evolving superstatistical parameters* q_t *(top) and* a_t *(bottom).*

Optimum values for the two control parameters p_{min} and R can be found systematically by generating synthetic data sets that resemble the actual data and by quantifying how well the known superstatistical parameter changes of (q_t, a_t) are reconstructed by the method. The values for p_{min} and R given in the paper have been obtained in this way.

If the dynamics of the superstatistical parameters (q_t, a_t) is entirely unknown, the generation of realistic synthetic data sets is impossible. In such cases, the choice of the control parameters p_{min} and R can be viewed as a Bayesian

model selection problem. Using measured data only, the plausibility of a given set (p_{min}, R) can then be quantified by the global marginal likelihood L: For each time step t, the momentary marginal likelihood L_t is obtained by integrating the likelihood function over the (q_t, a_t)-plane. This integral can be evaluated readily within our grid based approach. The global marginal likelihood L is then given by the product of all L_t. The optimal set of control parameters, given the measured data, can then be found by maximizing L.

Supplementary Note 6

Persistence time and cell speed

In a superstatistical data analysis, the parameters q_t and a_t used in this work can be replaced by different variables that may be more appropriate for specific questions. For example, persistence can also be quantified by a persistence time τ_t, defined as the half decay time of the velocity correlations. Assuming $0 < q_t < 1$, and measuring the correlation time in units of the sampling time, τ_t is monotonically increasing with q_t, according to

$$\tau_t = \frac{\log(1/2)}{\log(q_t)}.$$

Furthermore, rather than investigating superstatistical parameter correlations between q_t and a_t, new insights can also be gained from the 'inter-level' correlations between superstatistical parameters and directly measured variables, such as the momentary cell speed u_t.

The relationship between cell speed, activity and cell persistence can be seen most easily in the case of a homogeneous AR-1 process in 2D. Assuming $0 \leq q_t < 1$, the momentary speed u_t is then Rayleigh distributed with the most probable value $\hat{u}_t$ being given by

$$\hat{u}_t = \frac{a_t}{\sqrt{1 - q_t^2}}.$$

In a hypothetical situation with $a = constant$, there is always a positive correlation between $\hat{u}_t$ and q_t (or, equivalently, between u_t and τ_t). However, in the case of migrating cells, the situation is more complicated as the activity a_t is also a strongly time-varying quantitiy that may be correlated positively or negatively with the persistence q_t.

For example, Miauri et al. have recently reported an increase of the momentary persistence time τ_t with the momentary cell speed u_t for a variety of different cell types migrating on fibronectin-coated surfaces (Ref.[26] in the main manuscript). From our cell migration experiements on fibronectin-coated plastic, we obtain a similar dependence of τ_t on u_t, including the saturation of persistence time for larger cell speeds (Supplementary Figure 10).

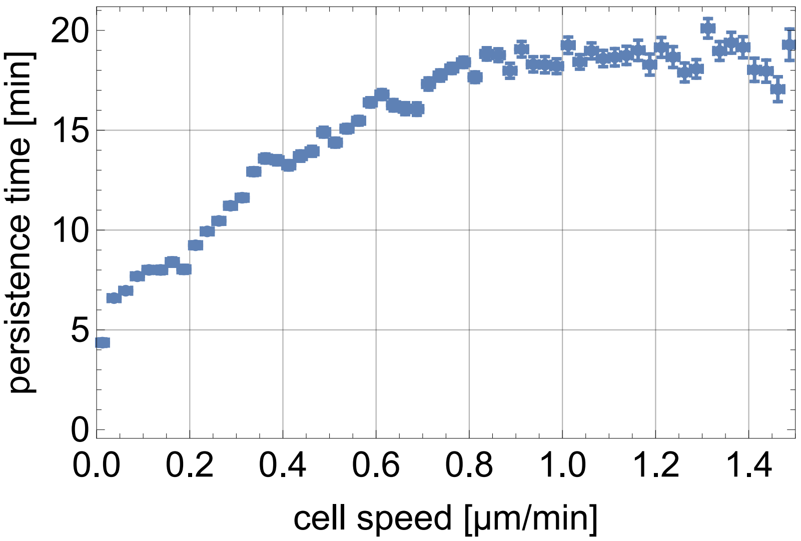

Supplementary Figure 10: ***Persistence time versus momentary speed for MDA-MB-231 cells on fibronectin-coated plastic.*** *Horizontal bars indicate the bins of cell speed, vertical bars the standard error of the mean persistence time.*

On uncoated plastic, where persistence is considerably lower, τ_t also increases with u_t, but without clear signs of saturation (Supplementary Figure 11).

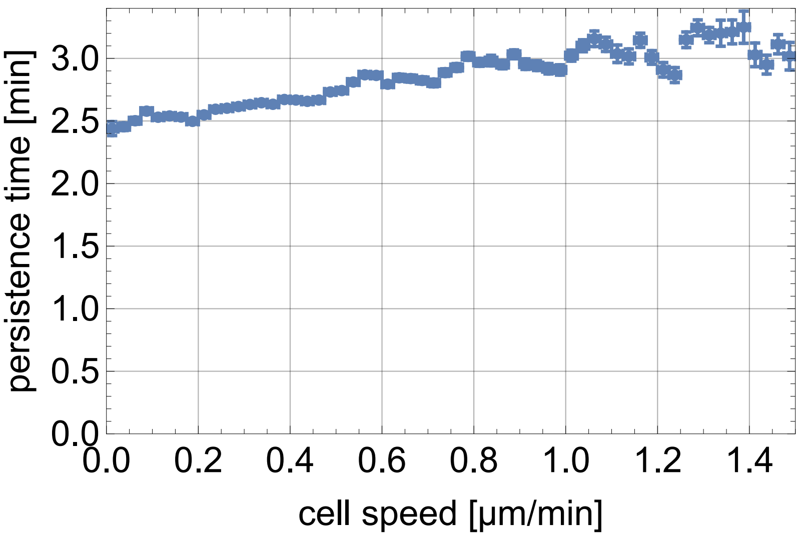

Supplementary Figure 11: ***Persistence time versus momentary speed for MDA-MB-231 cells on uncoated plastic.*** *Horizontal bars indicate the bins of cell speed, vertical bars the standard error of the mean persistence time.*

For cell migration in collagen, we find only a weak dependence of τ_t on u_t (Supplementary Figure 12).

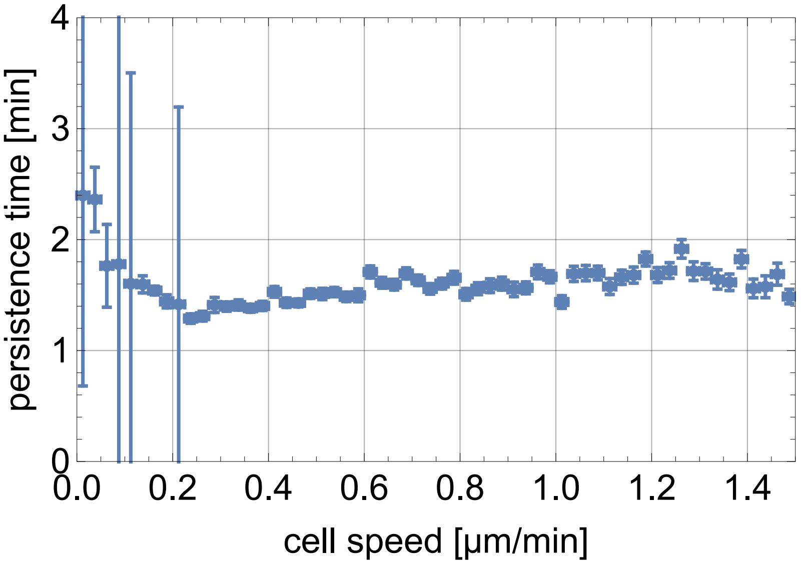

Supplementary Figure 12: ***Persistence time versus momentary speed for MDA-MB-231 cells in collagen.*** *Horizontal bars indicate the bins of cell speed, vertical bars the standard error of the mean persistence time.*
